# Surface Passivation and Energetic Modification Suppress Nonradiative Recombination in Perovskite Solar Cells

**DOI:** 10.1007/s40820-022-00854-0

**Published:** 2022-04-19

**Authors:** Wei Dong, Wencheng Qiao, Shaobing Xiong, Jianming Yang, Xuelu Wang, Liming Ding, Yefeng Yao, Qinye Bao

**Affiliations:** 1 Shanghai Key Laboratory of Magnetic Resonance, East China Normal University, Shanghai, 200241 People’s Republic of China; 2 School of Physics and Electronic Science, East China Normal University, Shanghai, 200241 People’s Republic of China; 3 Center for Excellence in Nanoscience (CAS), Key Laboratory of Nanosystem and Hierarchical Fabrication (CAS), National Center for Nanoscience and Technology, Beijing, 100190 People’s Republic of China; 4 Collaborative Innovation Center of Extreme Optics, Shanxi University, Taiyuan, 030006 Shanxi People’s Republic of China

**Keywords:** Passivation, Solid-state NMR, Charge transport, Perovskite solar cell

## Abstract

Surface passivation via post-treatment is an important strategy for improving power conversion efficiency and operational stability of perovskite solar cells. However, so far the interaction mechanisms between passivating additive and perovskite are not well understood. Here, we report the atomic-scale interaction of surface passivating additive 2,2-difluoroethylammonium bromine (2FEABr) on the MAPbI_3_. It is found that the bulky 2FEA^+^ cations tend to distribute at film surface, while the Br^−^ anions diffuse from surface into bulk. A combination of ^19^F, ^207^Pb, and ^2^H solid-state NMR further reveal the Br^−^ anions’ partial substitution for the I^−^ sites, the restricted motion of partial MA^+^ cations, and the firmed perovskite lattices, which would improve charge transport and stability of the perovskite films. Optical spectroscopy and ultraviolet photoelectron spectroscopy demonstrate that the 2FEABr induced surface passivation and energetic modification suppress the nonradiative recombination loss. These findings enable the efficiency of the *p*-*i*-*n* structured PSC significantly increasing from 19.44 to 21.06%, accompanied by excellent stability. Our work further establishes more knowledge link between passivating additive and PSC performance.

**Supplementary Information:**

The online version contains supplementary material available at 10.1007/s40820-022-00854-0.

## Highlights

- The partial substitution of Br− on I-sites, and the restricted motion of MA^+^ cations in correlation with suppressed electron-phonon coupling promote charge transport.
- The perovskite parent lattice of 2FEABr-treated perovskites was firmed, and the difficulty degree for A-site MA^+^ cations running out of the inorganic framework was thus enhanced.
- The efficiency was enhanced from 19.44% to 21.06%, accompanied with excellent stability.

**Supplementary Information:**

The online version contains supplementary material available at 10.1007/s40820-022-00854-0.

## Graphical abstract

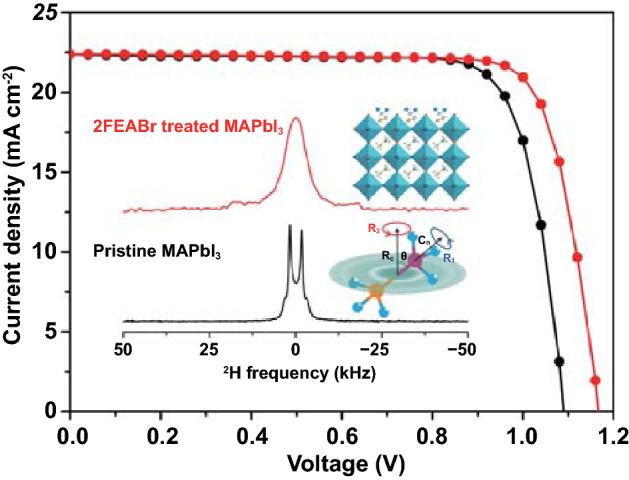

## Introduction

Organic–inorganic hybrid perovskites are promising semiconductors suitable for next generation solar energy technology owing to the excellent photoelectronic merits of high light absorption coefficient [1, 2], long electron–hole diffusion length [3, 4], tunable bandgap [5–7], and small exciton binding energy [8, 9]. Attributed to the implements of material compositional engineering [10–12], solvent engineering [13–15] and device architecture design [16–18], the certified power conversion efficiencies (PCE) of perovskite solar cells (PSCs) have reached 25.7% within a decade of efforts [19]. However, the devices are suffering from significant nonradiative recombination losses, severely limiting their thermodynamic efficiency [20–23].

Tremendous experimental and theoretical works have demonstrated that defect passivation on polycrystalline perovskite films is a promising strategy for both suppressing carrier recombination and stabilizing perovskite structure [24–31]. The dominated defects locate at grain boundaries and on surface of perovskite films, and the surface defect densities are approximately two orders much than that in the bulk [32]. Among the various passivating additives reported, it has been well-established that ammonium salts can effectively reduce the defects by bonding with the Pb-I antisites or cation vacancies, thereby increasing the device performance [25, 33, 34]. Surface post-treatment is a commonly used approach to introduce these passivators into perovskite semiconductors. For instance, Hagfeldt and co-workers recently shown that a mixed-ammonium salt formamidinium bromide (FABr) via reacting with the excess PbI_2_ resulted in a high-quality perovskite surface with less defects and an improved 23.5% efficiency in *n*-*i*-*p* structural PSCs [35]. Stranks and co-workers reported that the ammonium salt methylammonium tetrafluoroborate (MABF_4_) could achieve the small nonradiative recombination with a reduction in first-order recombination rate in PSCs [36]. However, the atomic-scale spatial distribution of ammonium salt passivator in perovskite film, and the effect of passivator on perovskite lattice are unclear, which are critical to better understand the working mechanisms and thus provide guidance for designing more effective passivating additives towards high-performance PSCs.

In this work, we report the atomic-scale interaction of fluorinated ammonium salt 2,2-difluoroethylammonium bromine (2FEABr) surface passivating additive on hybrid perovskite MAPbI_3_ for photovoltaics. It is found that the bulky 2FEA^+^ cations tend to distribute at film surface, while the Br^−^ anions diffuse from surface into bulk. A combination of ^19^F, ^207^Pb, and ^2^H solid-state nuclear magnetic resonance (NMR) technologies are carefully employed to systematically explore the subtle interactions between 2FEABr and MAPbI_3_. We demonstrate that the Br^−^ anions of 2FEABr partially substitute for the I^−^ sites in perovskite lattices, and the motion of partial MA^+^ cations is restricted in correlation with suppressed electron–phonon coupling, which are beneficial for suppressing nonradiative recombination. These findings are confirmed by the power conversion efficiency of the *p*-*i*-*n* structured PSC significantly increasing from 19.44 to 21.06%. Moreover, the unencapsulated device exhibits excellent thermal stability with maintaining over 95% of its initial efficiency at 340 K after 1000 h. The NMR signals confirm that the perovskite parent lattice is firmed after 2FEABr post-treatment and the degree of difficulty of the A^−^ site MA^+^ cations running out of the inorganic framework is thus enhanced, leading to a robust perovskite film. This work provides more insights into working mechanisms of passivating additive for highly efficient and stable PSCs.

## Experimental Section

### Materials

Methylammonium iodide (MAI) was purchased from Greatcell Solar Company. Lead iodide (PbI_2_) was obtained from TCI. Phenyl-C61-butyric acid methyl ester (PCBM), Poly(triarylamine) (PTAA), Bathocuproine (BCP) and 2,3,5,6-Tetrafluoro-7,7,8,8-tetracyanoquinodimethane (F4-TCNQ) were brought from Xi’an Polymer Light Technology Corp. The anhydrous solvents including *N*, *N*-dimethylformamide (DMF), chlorobenzene (CB) and isopropanol (IPA) were purchased from Sigma-Aldrich. 2,2-difluoroethylammonium bromine (2FEABr) was synthesized following the procedure from a literature [37].

### Device Fabrication

The patterned ITO substrates (NSG 10, Nippon sheet glass) were cleaned by sequentially sonication with detergent, DI water, acetone, ethanol and IPA for each 20 min, respectively. The cleaned ITO substrates were dried in an oven at 80 °C and treated with UV-ozone for 20 min. 2 mg mL^−1^ PTAA with 25 wt% F4TCNQ in CB was spun onto the ITO substrates at 5000 rpm for 30 s, and then annealed at 150 °C for 10 min. 100 μL DMF solvent was spin-coated to wet the PTAA layer surface at 4000 rpm for 10 s. The perovskite precursor was prepared by mixing 497.8 mg PbI_2_ and 159 mg MAI in a mixed solvent of 750 μL DMF and 85 μL DMSO. The perovskite precursor solution was spin-coated on PTAA layer at 4000 rpm for 30 s and 150 μL CB was dropped on the center of the film after 8 s processing, and then the perovskite film was put onto a hotplate at 100 °C for 10 min. For the post-treatment process, the synthesized 2FEABr was dissolved in IPA with the different concentrations (1, 2, 3, 5 mg mL^−1^) and spin-coated onto the perovskite surface at 6000 rpm for 30 s, followed by annealing at 60 °C for 10 min. 20 mg mL^−1^ PCBM in CB was spin-coated onto the perovskite film at 1800 rpm for 40 s, and 0.5 mg mL^−1^ BCP in IPA was spin coated at 6000 rpm for 30 s to form an interfacial layer. Finally, 100 nm Ag electrode were thermally evaporated in a vacuum chamber at the pressure of 2 × 10^–4^ Pa with a shadow mask (aperture area: 0.05 cm^2^).

### Film Characterization

X-ray diffraction (XRD) patterns were recorded using a Rigaku Smart Lab diffractometer with Cu-K*α* radiation as the X-ray source. Atomic force microscopy (AFM) images were obtained from an atomic force microscope (Shimadzu, SPM-9700) in tapping mode. UV–vis absorption spectra were measured by a UV–vis spectrometer (Shimadzu 3600). Steady-state photoluminescence (PL) spectra were obtained using a fluorescence spectrometer (Edinburgh, FLS-980). Time-resolved photoluminescence (TRPL) was performed by a time-correlated single-photon counting (TCSPC) system. A picosecond laser with a wavelength of 485 nm (C10196, Hamamatsu) was employed to excite the films. X-ray photoelectron spectroscopy (XPS) and ultraviolet photoelectron spectroscopy (UPS) measurements were conducted in an ultrahigh vacuum surface analysis system equipped with SCIENTA R3000 spectrometer with a base pressure of 10^−10^ mbar. XPS was measured using the monochromatic Al Ka 1486.6 eV as excitation source, and UPS employed the He I 21.22 eV as the excitation source with an energy resolution of 50 meV. All spectra were calibrated by referring to Fermi level and Au 4 f_7/2_ position of the Ar^+^ ion sputter cleaned Au foil.

### Solid-State NMR Spectroscopy

^2^H and ^207^Pb static nuclear magnetic resonance (NMR) experiments were performed in a Bruker 300 MHz NMR spectrometer operating at 46.07 MHz for ^2^H and 62.73 MHz for ^207^Pb. A Bruker two-channel static PE probe with a home-made 2.5 mm coil was used to record the spectra. The ^2^H spectra were acquired using the solid echo sequence. The ^2^H pulse width was 2.5 μs at an RF field strength of 100 kHz. ^13^C and ^19^F magic angle spinning (MAS) NMR spectra were recorded by a Brucker 500 MHz NMR spectrometer at 25 °C at 10 kHz. ^13^C chemical shifts were referenced to adamantane (38.5 ppm). ^19^F chemical shifts were referenced to pure CFCl_3_ (0 ppm) using NaF (− 224 ppm) as a secondary reference. The powders were scrapped from glass substrates and packed in a MAS rotor in a N_2_-filled glovebox before being transferred into the NMR probe.

### Device Characterization

The *J-V* curves of perovskite solar cells were characterized using a digital source meter (Keithley 2400) under Enlitech Solar Simulator (AM 1.5G, 100 mW cm^−2^) that calibrated by a standard silicon solar cell from NREL. The EQE spectra were obtained using an Enlitech QE-R system under AC mode. The Mott-Schottky curves were recorded at 1 kHz from an electrochemical workstation (CHI 650E). The electronic impedance spectroscopy (EIS) measurements were performed at a bias of 0.8 V in the dark by an impedance spectroscope (PGSTAT302N, Autolab) with the frequency ranging from 1 MHz to 1 Hz.

Space charge limited current (SCLC) measurement was applied to determine the electron trap density and mobility using the electron-only device with a structure of ITO/SnO_2_/MAPbI_3_/2FEABr/PCBM/BCP/Ag. The trap state density (*N*_*t*_) was determined by the onset of the trap filling limit voltage (*V*_TFL_) according to the equation of $V_{{{\text{TFL}}}} = \frac{{eN_{t} L^{2} }}{{2\varepsilon \varepsilon_{0} }}$, where *ε*_*0*_ is the permittivity of free space, *ε*_*r*_ is the relative permittivity of perovskite, and *L* is the thickness of perovskite layer. The electron mobility (*μ*_*e*_) is calculated by fitting the SCLC with the Motto-Gurney law of $J_{d} = \frac{9}{8}\varepsilon \varepsilon_{0} \mu \frac{{V^{2} }}{{L^{3} }}$, where *J* is the current density, and *V* is the base voltage.

## Results and Discussion

**Fig. 1:**
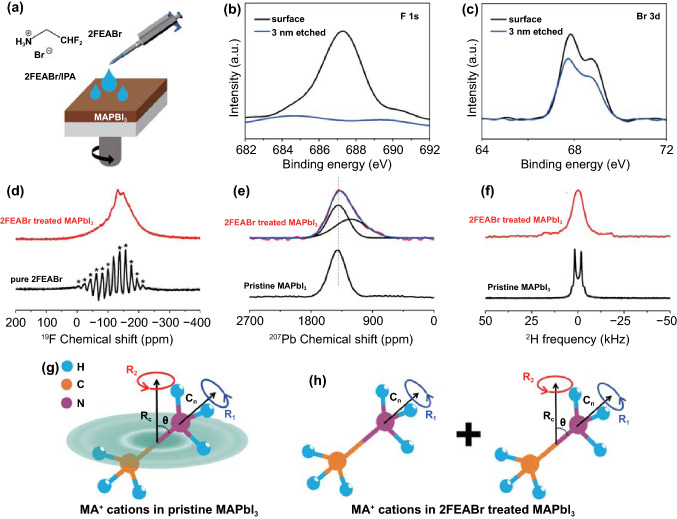
**a** Schematics of 2FEABr post-treatment process and molecule structure of 2FEABr. **b–c** F 1*s* and Br 3*d* XPS core level spectra of 2FEABr treated perovskite film before and after surface etching. **d** ^19^F MAS NMR. **e** ^207^Pb NMR. **f** ^2^H NMR. **g–h** Motion models of MA^+^ cations in pristine MAPbI_3_ and in 2FEABr treated perovskites

2FEABr solution at 2 mg mL^−1^ is spin-coated on MAPbI_3_ perovskite surface and annealed at 60 °C for 10 min. The surface post-treatment process and the 2FEABr molecular structure are illustrated in Fig. 1a. The distribution of 2FEA^+^ cations and Br^−^ anions in the perovskite film is intuitively confirmed by XPS depth profiling analysis of F 1*s* and Br 3*d* core level spectra (Fig. 1b–c). The F 1*s* signal disappears after 3 nm etched from the film surface, while the Br 3*d* is clearly observed. It is thus concluded that the bulky 2FEA^+^ cations tend to distribute at surface, while the Br^−^ anions could diffuse from surface into the bulk. The incorporation of Br^−^ in perovskites has been demonstrated to enhance the carrier lifetime, which could improve the device optoelectronic performance [38, 39]. In addition, there is no clear change in perovskite XRD patterns before and after 2FEABr post-treatment (Fig. S1). To obtain more details of the crystal structure, we also performed the gracing incidence X-ray diffraction (GIXRD) measurement (Fig. S2). No new diffraction peak is observed when the incident angle increased from 0.3 to 5°, indicating no low dimensional phase after 2FEABr treatment.

The ^19^F magic angle spinning (MAS) NMR spectroscopy is employed to study the environment of 2FEA^+^ cations in pure 2FEABr and 2FEABr treated MAPbI_3_ (Fig. 1d). For the pure 2FEABr, the ^19^F NMR signal shows a pronounced asymmetric peak centered at − 117.5 ppm with full width at half maximum (FWHM) of only 6 ppm. The spike like signals marked by the asterisks are the sideband manifolds. For 2FEABr treated MAPbI_3_, the ^19^F NMR appears as a wide peak with FWHM of 119 ppm. Intriguingly, the sideband manifolds are not observed here, meaning that the local environments of ^19^F in perovskite are quite different from those in the pure 2FEABr [40]. This pronounced change is attributed to the enhancement of dipolar coupling, indicating that the 2FEA^+^ cations bond with Br^−^ and interact with surface Pb-I antisite defects through electrostatic interaction [41–43]. Figures 1e and S4 present the ^207^Pb NMR spectra of the pristine and 2FEABr treated perovskites. The ^207^Pb spectra of the pristine MAPbI_3_ shows a line shape of a symmetrical broad peak centered at 1409 ppm, and the ^207^Pb NMR signal of the 2FEABr treated MAPbI_3_ shows a clear asymmetric peak, consisting of two components centered at 1401 and 1219 ppm, respectively. The former corresponds to [PbI_6_]^4−^ structure of the pristine MAPbI_3_, and the latter is probably attributed to [PbI_6−*x*_Br_*x*_]^4=^ species, where the Br^−^ anions have partially substituted for the I^−^ sites [44]. In principle, the Br substitution will cause the formation of the disordered structures in the lattice. However, no clear evidence of such disordered structures was observed in the XRD results (Fig. S1), which means that the ^207^Pb NMR is a more sensitive technique to probe the disordered structures in the crystal lattice of perovskites.

Attributing to the subtle interaction between the embedded organic cations and inorganic frameworks in perovskite lattice, the molecular motion of the embedded organic cation is often very sensitive to the lattice change. The lattice changes thus can be monitored by observing the molecular motion change of the embedded cations [45, 46]. Figure 1f and S5 show the corresponding ^2^H NMR spectra of the pristine and 2FEABr treated MAPbI_3_. The ^2^H NMR spectra of the pristine MAPbI_3_ has a typical Pake pattern with a splitting of 4 kHz between the two horns. A new complicated line shape consisting of a narrow single peak and a broad characteristic component appears in 2FEABr treated MAPbI_3_, whereas the original Pake line shape completely vanishes. The motional geometry of the MA^+^ cations is further analyzed by the pattern simulation based on the different motion models (Figs. S6 and S7). The Pake line shape in the ^2^H NMR spectra of the pristine MAPbI_3_ indicates that the MA^+^ cations undergo a double rotation, that is, a rotation of the ND_3_ group about the C–N axis (*R*_1_ axis) and a rotation of C–N axis about *R*_2_ axis (Fig. 1g). The complicated line shape of 2FEABr treated MAPbI_3_ can be decomposed into a hump component together with a broad component (detailed information in Fig. S7). The broad component (red) with a splitting of about 40 kHz is attributed to completely restricted rotation of C–N axis about *R*_2_ axis in partial MA^+^ cations (the left model in Fig. 1h). Meanwhile, the emerged hump component (blue) with FWHM of 5 kHz points to the inconsistency of rotation angle of MA^+^ cations, indicating diverse isotropic motion components of MA^+^ cations in lattice (the right model in Fig. 1h). The restricted motion of the partial MA^+^ cations correlates with suppressed electron–phonon coupling, which is expected to suppress nonradiative recombination and enhance charge mobility [47, 48].

**Fig. 2:**
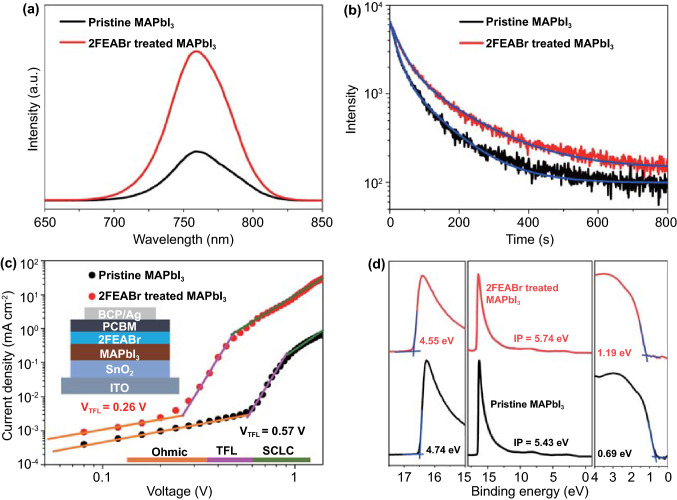
**a** Steady-state PL spectra of pristine and treated perovskite films. **b** Time-resolved PL decay. **c** Dark J-V curves of electron-only devices. **d** UPS spectra

The root mean square (RMS) roughness of the 2FEABr treated perovskite films measured via AFM decreases from 20.46 to 17.74 nm, in favor of the perovskite contacting with the top charge transport layer (Fig. S8). The UV–vis absorption spectra display an obvious blue shift due to Br^−^ doped into the perovskite lattice (Fig. S9). We then perform PL spectra to investigate the charge dynamics of the films. The improved PL intensity provides the solid evidence that 2FEABr helps to suppress nonradiative recombination by Br^−^ substitution, passivate defect and restrict MA^+^ motion (Fig. 2a). From the fitted time-resolved PL decays (Fig. 2b and Table S2), the 2FEABr treated perovskite films exhibit longer carrier lifetime (*τ*_1_ = 32.3 ns, *τ*_2_ = 145.6 ns) than the pristine films (*τ*_1_ = 20.5 ns, *τ*_2_ = 101.8 ns), further confirming that the 2FEABr suppresses the charge recombination in the perovskite films (Fig. 2b). We evaluate the charge trap density and mobility of the 2FEABr treated perovskite films using the space-charge-limited current (SCLC) method. As determined from the dark J-V curves of electron-only device with a structure of ITO/SnO_2_/MAPbI_3_/2FEABr/PCBM/BCP/Ag, the calculated electron trap densities dimmish from 1.18 × 10^16^ cm^−3^ for the pristine to 5.39 × 10^15^ cm^−3^ for the 2FEABr treated perovskite (Fig. 2c). The electron mobility also increases from 4.33 × 10^–2^ to 6.45 × 10^–1^ cm^2^ V^−1^ s^−1^, nearly one order of magnitude, boosting the charge transport. Moreover, we characterize surface electronic structures of the perovskite films by ultraviolet photoelectron spectroscopy (UPS) (Fig. 2d). The secondary electron cutoff region determines the work function (WF), where the WF of the perovskite film decreases from 4.74 to 4.55 eV after 2FEABr post-treatment, which could further improve the electron extraction efficiency at perovskite/the top electron transport layer interface. In addition, the Fermi level (*E*_*F*_) position with respect to the valence band maximum (VBM) shifts away from 0.69 to 1.19 eV, indicating the formation of more *n*-type perovskite surface that matches with the top electron transport layer [49, 50]. Figure S10 presents the corresponding energy level diagram.

**Fig. 3:**
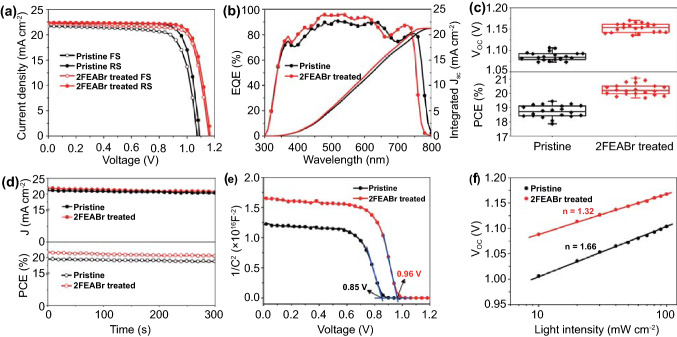
**a** *J-V* characteristics of PSC devices under forward and reverse scans. **b** EQE spectra with integrated photocurrent. **c** Statistics of *V*_oc_ and PCE. **d** Steady-state photocurrent and power output at the maximal power point. **e** Mott-Schottky plots. **f** Dependence of *V*_oc_ on light intensity

To confirm the effect of 2FEABr surface treatment on device performance, we fabricate the *p*-*i*-*n* planar heterojunction PSC with an architecture of ITO/PTAA:F4TCNQ/perovskite/2FEABr/PCBM/BCP/Ag as illustrated in Fig. S12. The dependence of the device efficiency on 2FEABr concentration is carefully evaluated (Fig. S13 and Table S3), and the optimal concentration that produces the champion device is 2 mg mL^−1^. Figure 3a displays the current density–voltage (*J-V*) curves of the devices under a simulated AM 1.5 G light illumination at 100 mW cm^−2^. The pristine device has a typical PCE of 19.44% with an open circuit voltage (*V*_oc_) of 1.090 V, a short current density (*J*_sc_) of 22.38 mA cm^−2^, and a fill factor (FF) of 0.797. The champion device with 2FEABr yields a significantly improved PCE of 21.06% with a *V*_oc_ of 1.166 V, a *J*_sc_ of 22.39 mA cm^−2^, and an FF of 0.807 (Table 1). Clearly, the enhanced device efficiency originates from the increased *V*_oc_ and FF.

**Table 1:** Photovoltaic parameters of the champion PSCs with and without 2FEABr under forward scan (FS) and reverse scan (RS)

	*V*_oc_ (V)	*J*_sc_ (mA cm^−2^)	FF	PCE (%)	HI
Pristine RS	1.090	22.38	0.797	19.44	0.095
Pristine FS	1.073	21.72	0.753	17.59	
2FEABr treated RS	1.166	22.39	0.807	21.06	0.041
2FEABr treated FS	1.158	22.23	0.785	20.20	

Figure 3b shows the external quantum efficiency (EQE) spectra and the integrated photocurrent densities. The absorption edge of the 2FEABr treated device is blue shifted about 20 nm, in consistent with the UV–vis absorption spectra shift of the perovskite film observed in Fig. S9, which leads to the nearly same photocurrents of the devices before and after 2FEABr treatment. The integrated current densities obtained from EQE are 21.28 mA cm^−2^ for the pristine device and 21.20 mA cm^−2^ for the 2FEABr treated device, respectively, which are comparable with *J*_sc_ in the *J-V* curves. The statistical charts of the photovoltaic parameters PCE, *V*_oc_ and FF, demonstrate the good reproducibility of the device performance (Figs. 3c and S14). As shown in Fig. 3d, the 2FEABr treated device exhibits a stabilized power output of 20.63% and a steady-state current density of 21.05 mA cm^−2^ at the maximum power point (MPP) under continuous illumination for 300 s.

To better understand the enhanced *V*_oc_, we carry out capacitance–voltage (*C-V*) curves to directly compare the built-in potential (*V*_bi_) of the devices by the Mott-Schottky equation of $\frac{1}{{C^{2} }} = \frac{{2\left( {V_{{{\text{bi}}}} - V} \right)}}{{A^{2} e\varepsilon_{0} \varepsilon N_{A} }}$, where *A* is device area, *ε* is relative permittivity, *ε*_0_ is vacuum permittivity, and *N*_*A*_ is carrier concentration. The *V*_bi_ of the devices largely increases from 0.85 to 0.96 V after 2FEABr treatment (Fig. 3e), in agreement with the *V*_oc_ enhancement. We ascribe the increased flat band potential to the reduced work function and defect passivation of the perovskite surface. As shown in Fig. 3f, the slope of the dependence of *V*_oc_ on light intensity (*P*) is conducted to explore the degree of the trap-assisted recombination via the equation of ${\text{V}}_{oc} = \frac{nkT}{{qln\left( P \right)}}$, where *k* is Boltzmann constant, and *T* is absolute temperature. Compared to the slope of 1.66 kT/*q* for the pristine device, the slope deceases to 1.32 kT/*q* for the 2FEABr treated device, demonstrating that the trap-assisted recombination is effectively suppressed. These results accord with the increased recombination resistance (*R*_rec_) fitted in the electrical impedance spectroscopy (Fig. S15 and Table S4).

**Fig. 4:**
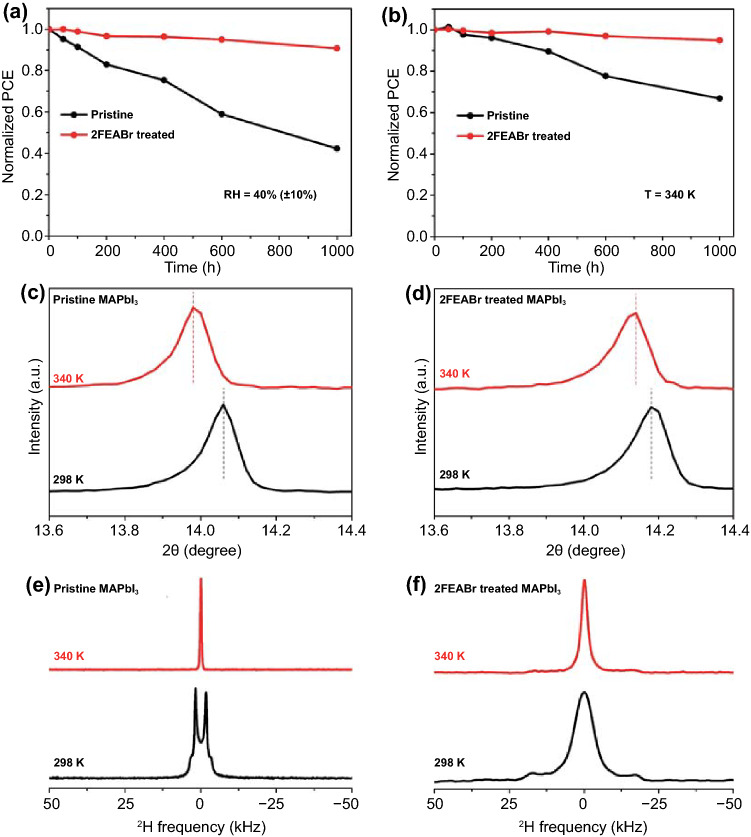
**a-b** Comparison of efficiency decay of unencapsulated PSCs as stored under 40% (± 10%) RH and 340 K, respectively. **c–d** Magnified XRD (100) peaks for pristine MAPbI_3_ and 2FEABr treated MAPbI_3_ at 298 K and 340 K, respectively. **e–f** ^2^H NMR spectra of pristine MAPbI_3_ and 2FEABr treated MAPbI_3_

The device stability before and after 2FEABr treatment is comparatively explored. Under ambient air in 40 ± 10% relative humidity (RH) at room temperature, the unencapsulated device retains over 90% of its initial efficiency after 1000 h storage, whereas the pristine device losses 60% of its initial value (Fig. 4a), mainly attributed to the 2FEABr coated on the perovskite film surface acting like moisture protection layer and the more hydrophobicity of the 2FEABr treated perovskite film confirmed by the increased water contact angel (Fig. S16). The device thermal stability is also significantly enhanced at 340 K in a nitrogen-filled glovebox, where the unencapsulated device maintains over 95% of its initial efficiency after 1000 h storage, and the pristine device losses nearly 40% of its initial value (Fig. 4b). In addition, we measure the XRD patterns of the films before and after 500 h calcination at 340 K (Fig. S17). It is clear that the pristine MAPbI_3_ significantly degrades to PbI_2_, while the 2FEABr treated film has negligible degradation under the same conduction. To gain more insight on the much-enhanced thermal stability of the device, the effect of 2FEABr on the perovskite lattice is explored via a combination of XRD and ^2^H NMR measurements. The XRD (211) characteristic peaks at 23.5° disappear when being heating at 340 K for both the pristine and 2FEABr treated perovskite films (Fig. S18), indicating a phase transition from tetragonal to cubic phase (Fig. S19) [51]. However, the XRD (100) peak of the 2FEABr treated MAPbI_3_ has the smaller shift of 0.04° than 0.08° of the pristine film after heating at 340 K (Fig. 4c–d), meaning less lattice expansion. Figure 4e shows the ^2^H NMR spectra of the pristine MAPbI_3_ at 298 and 340 K, respectively. For the pristine MAPbI_3_, the line shape completely changes from Pake doublets (298 K) to a narrow single peak (340 K), implying the reorientation dynamics of MA^+^ cations transformation from anisotropic into isotropic motion. For comparison in Fig. 4f, in the 2FEABr treated perovskites the original line shape of the ^2^H NMR spectra does not completely turn into a narrow single peak, which indicates that the dynamics of MA^+^ is still anisotropic and the lattice expansion is slight at the elevated temperature of 340 K. We conclude that the apparent NMR signal change results from the change of the cation dynamics due to the phase transition in MAPbI_3_, and the perovskite parent lattice is firmed after 2FEABr post-treatment and thus the degree of difficulty of the A-site MA^+^ cations running out of the inorganic framework is enhanced, leading to a robust perovskite film [52, 53].

## Conclusion

In summary, we have investigated the subtle interactions of ammonium salt passivating additive 2FEABr and hybrid perovskite MAPbI_3_ for photovoltaics. It is found that the bulky 2FEA^+^ cations tend to distribute at surface, while the Br^−^ anions diffuse from surface into the bulk. SS-NMR further demonstrates that the atomic-scale information on the Br^−^ anions’ partial substitution for the I^−^ sites, the restricted motion of partial MA^+^ cations, and the firmed perovskite lattices in the 2FEABr treated perovskites, which boost the charge transport and stability of the perovskite film. Meanwhile, the 2FEABr induced surface passivation and energetic modification suppress the nonradiative recombination loss. These findings are confirmed by the efficiency of the *p*-*i*-*n* structured device significantly increasing from 19.44 to 21.06%, accompanied by excellent stability. Our work provides more insights into the working mechanisms between passivating additive and perovskite towards highly efficient and stable PSCs.

## Supplementary Information

Below is the link to the electronic supplementary material.

Supplementary file1 (PDF 823 kb)
