# Supplementary material for: Surface Passivation and Energetic Modification Suppress Nonradiative Recombination in Perovskite Solar Cells
Source: Nanomicro Lett. 2022 Apr 19;14:108. doi: 10.1007/s40820-022-00854-0 (PMC9018932; doi:10.1007/s40820-022-00854-0)
Supplement: Supplementary file 1 — Supplementary file1 (PDF 823 kb) [file 40820_2022_854_MOESM1_ESM.pdf]

Supporting Information for

Surface Passivation and Energetic Modification Suppress Nonradiative Recombination in Perovskite Solar Cells

Wei Dong[1], Wencheng Qiao[1], Shaobing Xiong[2], Jianming Yang[2], Xuelu Wang[1], Liming Ding[3,*], Yefeng Yao[1,*], Qinye Bao[2,4,*]

[1]Shanghai Key Laboratory of Magnetic Resonance, East China Normal University, Shanghai 200241, P. R. China

[2]School of Physics and Electronic Science, East China Normal University, Shanghai 200241, P. R. China

[3]Center for Excellence in Nanoscience (CAS), Key Laboratory of Nanosystem and Hierarchical Fabrication (CAS), National Center for Nanoscience and Technology, Beijing 100190, P. R. China

[4] Collaborative Innovation Center of Extreme Optics, Shanxi University, Taiyuan, Shanxi 030006, P. R. China

* Corresponding authors. E-mail: ding@nanoctr.cn (Liming Ding), yfyao@phy.ecnu.edu.cn (Yefeng Yao), qybao@clpm.ecnu.edu.cn (Qinye Bao)

Supplementary Figures and Tables

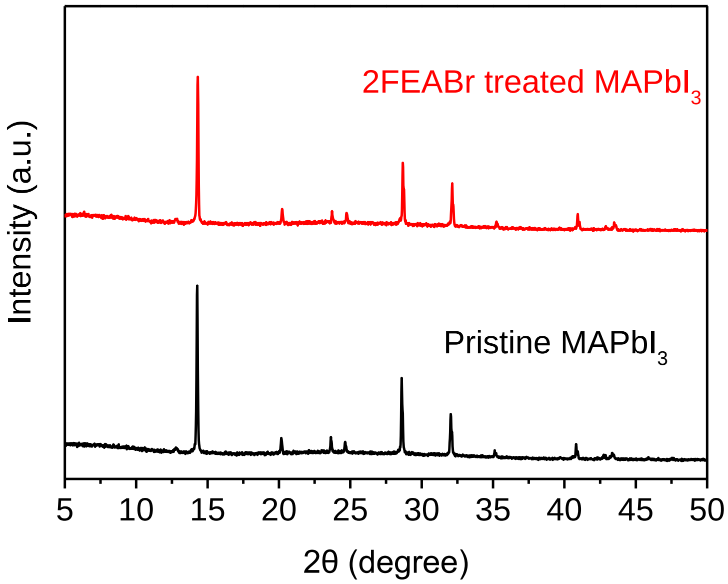

Fig. S1 XRD patterns of pristine and 2FEABr treated perovskite films

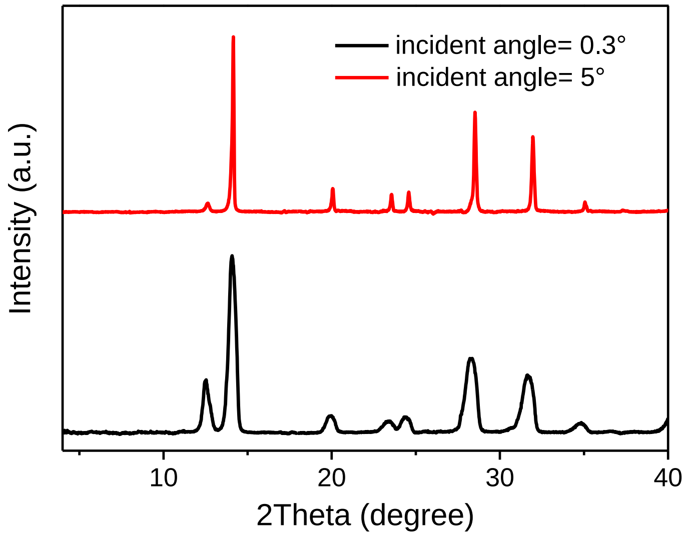

Fig. S2 GIXRD patterns of 2FEABr treated $MAPbI_3$ film

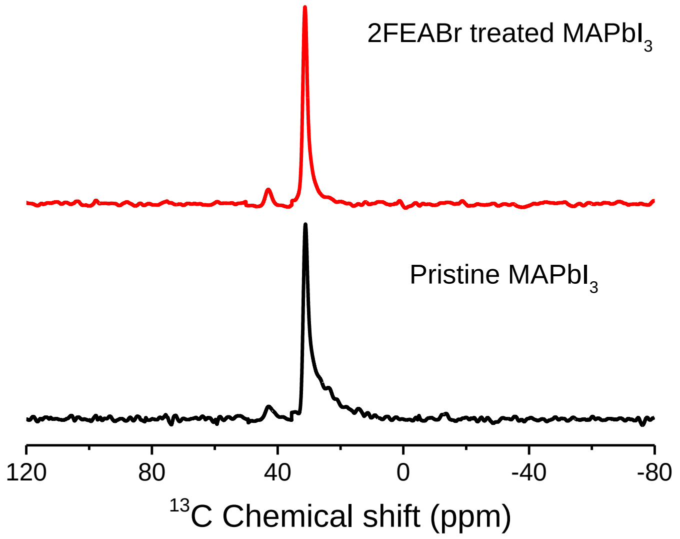

Fig. S3 ^{13}C MAS SS-NMR spectra of pristine and 2FEABr treated $MAPbI_3$

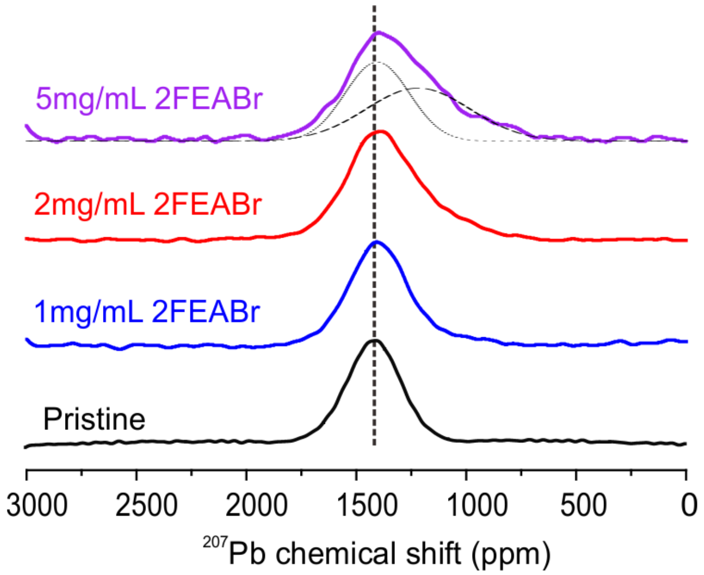

Fig. S4 ^{207}Pb NMR spectra of $MAPbI_3$ treated with different 2FEABr concentrations

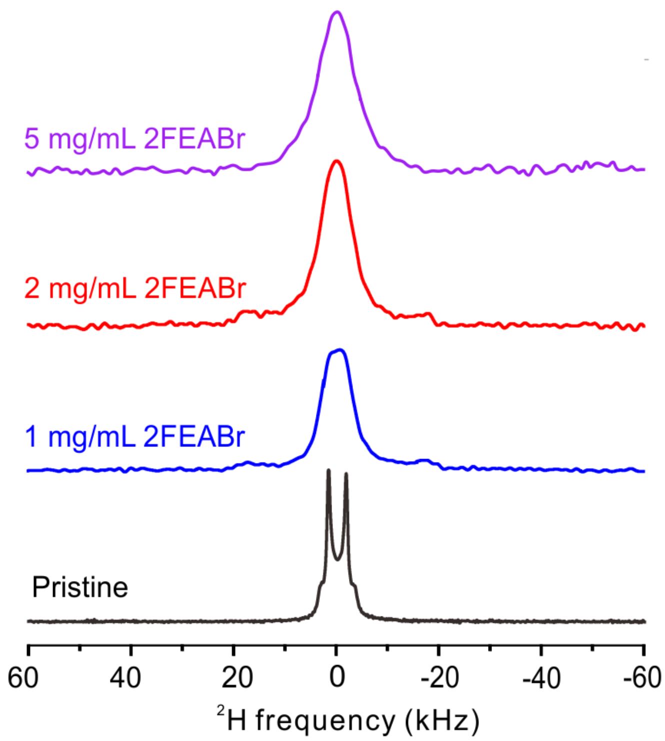

Fig. S5 ^{2}H NMR spectra of $MAPbI_3$ treated with different 2FEABr concentrations

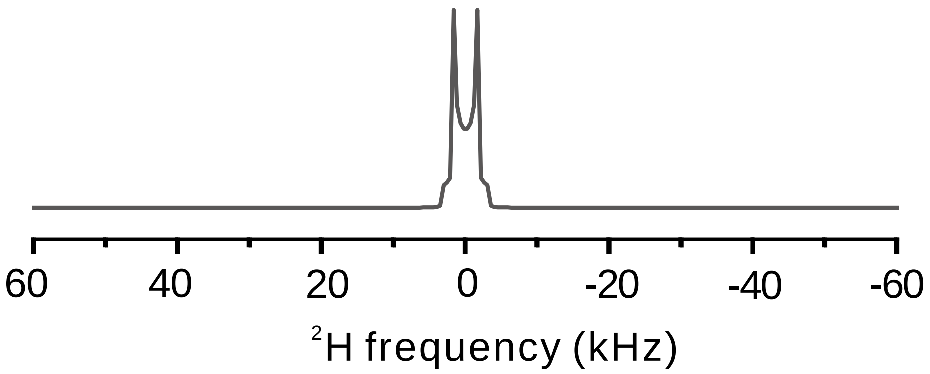

Fig. S6 The corresponding pattern simulation of Pake line shape in ^{2}H NMR spectrum of pristine $MAPbI_3$ sample. The inclined angle θ is the angle between C_n axis and R_c axis in the model in Figure 1g. The θ value obtained from the pattern simulation is 58.9°

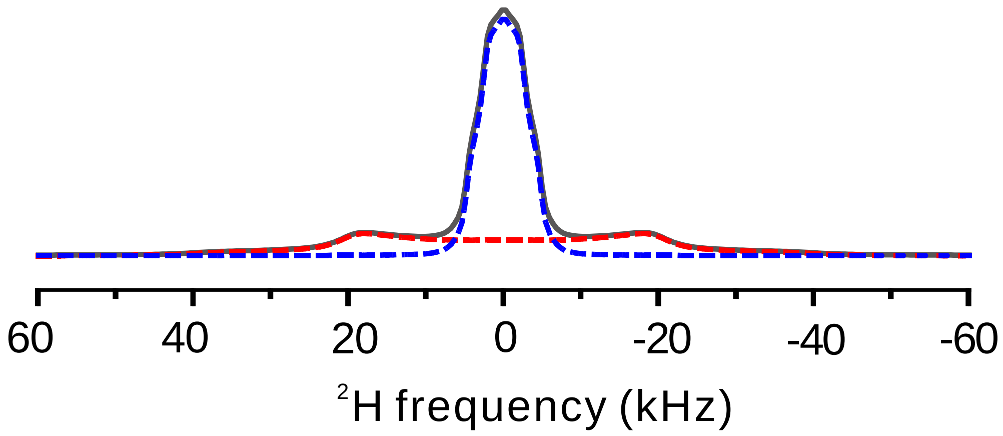

Fig. S7 The corresponding pattern simulation of the line shape of ^{2}H NMR spectrum of 2FEABr treated $MAPbI_3$

The interval of θ can be estimated from the Gaussian distribution:

$$f(\theta) = \frac{1}{\sigma\sqrt{2\pi}} \exp\,[\frac{(\theta - \theta_0)^2}{2\theta^2}]$$

Where the θ values are assumed to be distributed about a mean value θ_0 (58.9°) according to the Gaussian distribution with standard deviation σ. The obtained σ values from the simulation results are summarized in **Table S1**.

Table S1 σ values obtained from the simulated θ distribution of MA^+ cations motion model

2FEABr concentrations	pristine	1 mg/mL	2 mg/mL	5 mg/mL
standard deviation σ (°)	0	7	13	20

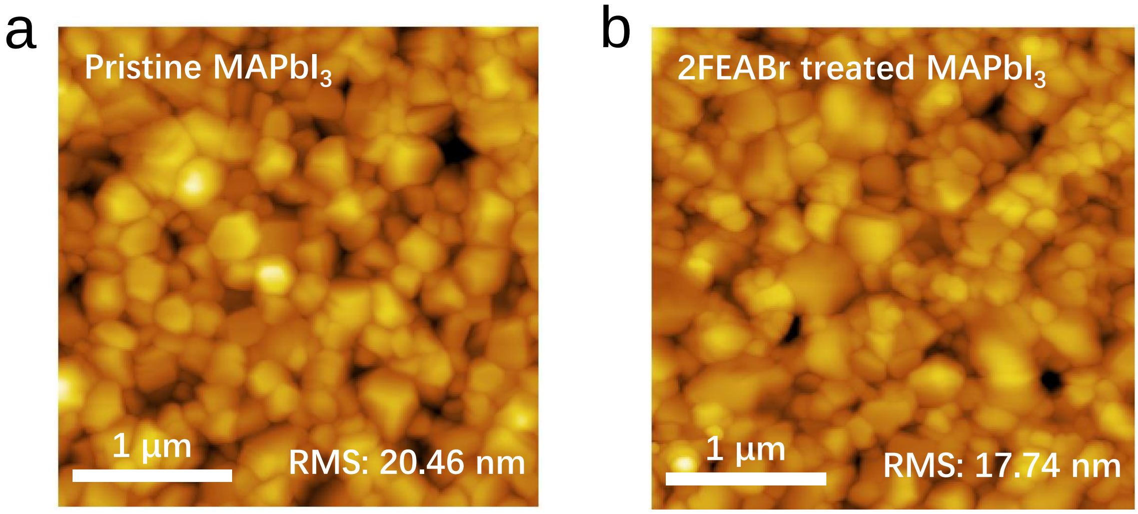

Fig. S8 AFM images of (**a**) pristine and (**b**) 2FEABr treated perovskite films

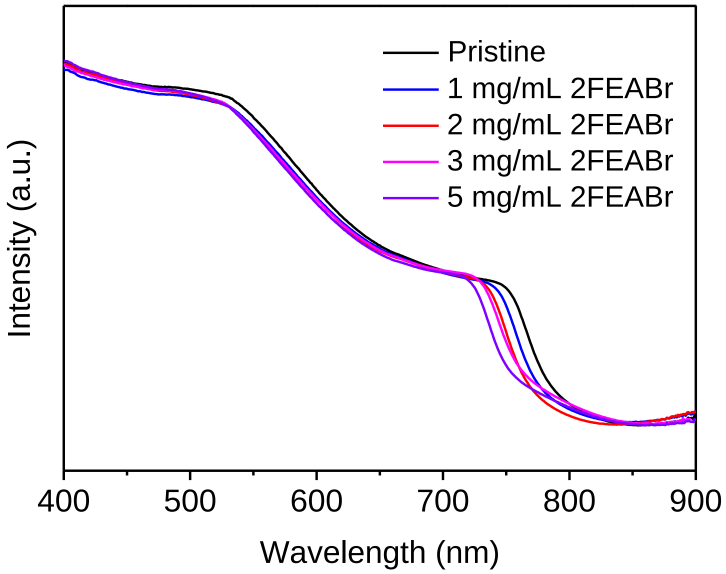

Fig. S9 UV-vis absorption spectra of $MAPbI_3$ films treated with different 2FEABr concentrations

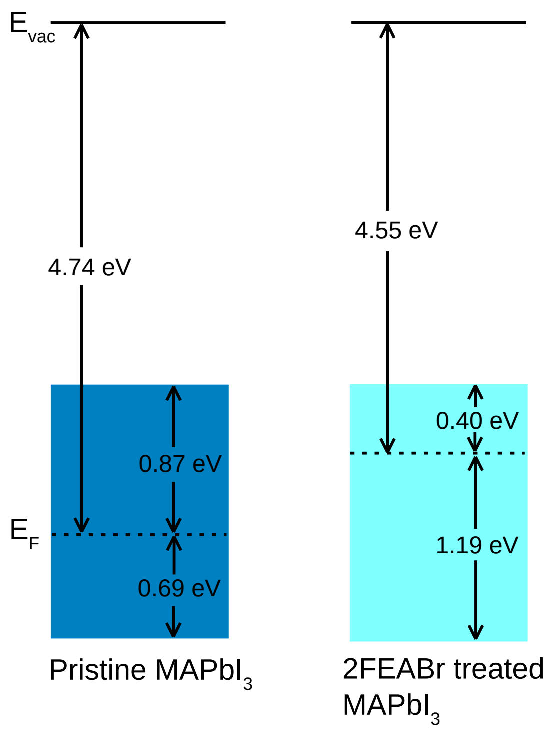

Fig. S10 Energy level diagram of pristine and 2FEABr treated perovskite film derived from UPS spectra

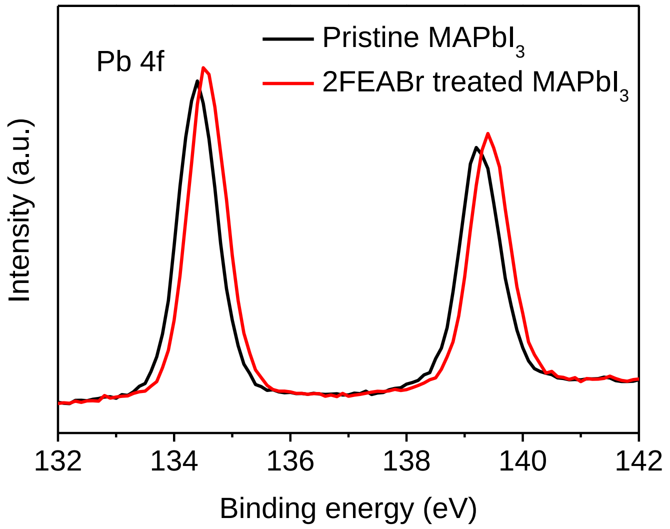

Fig. S11 Pb 4f XPS spectra of pristine and 2FEABr treated $MAPbI_3$ film

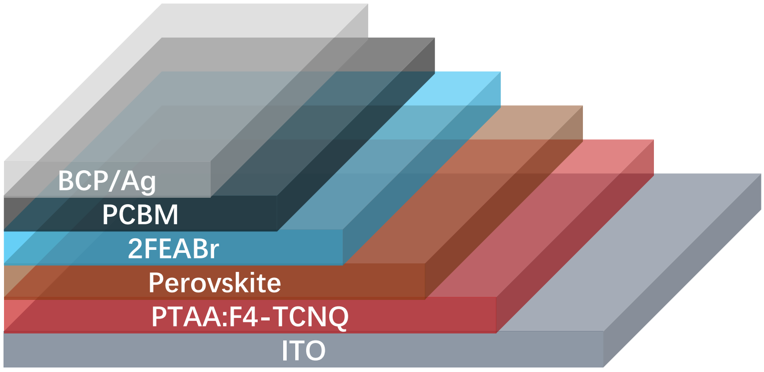

Fig. S12 Configuration of p-i-n structured PSC in this study

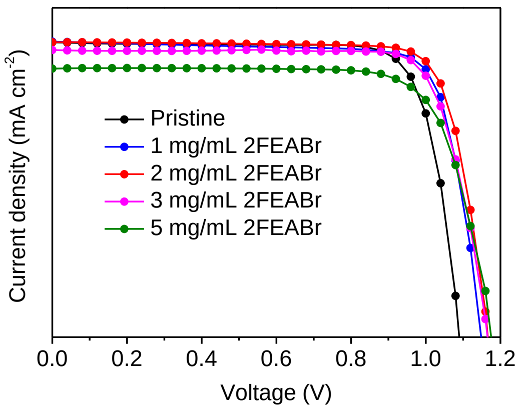

Fig. S13 J-V characteristic of champion devices treated with different 2FEABr concentrations

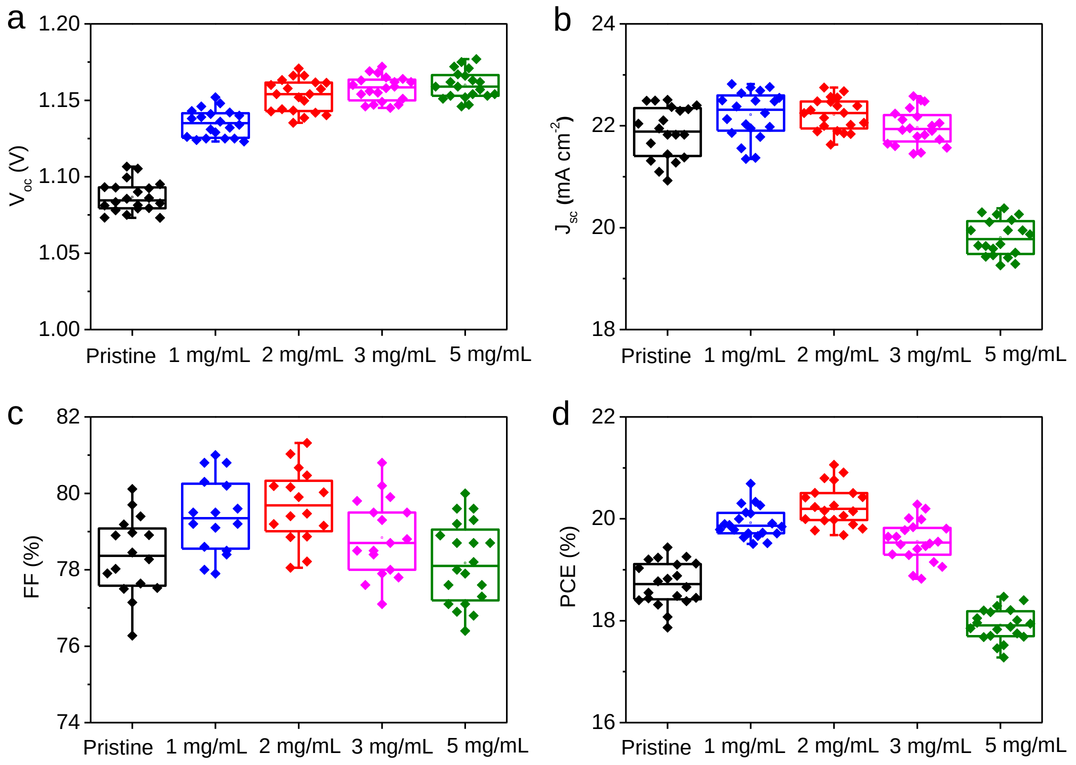

Fig. S14 Photovoltaic parameter distributions of the devices treated with different concentrations of 2FEABr: (**a**) V_{OC}, (**b**) J_{SC}, (**c**) FF, and (**d**) PCE

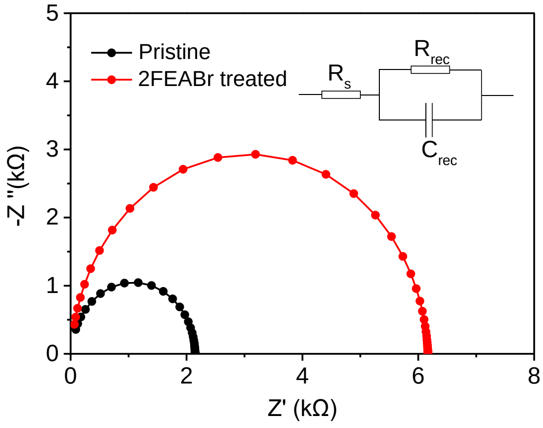

Fig. S15 Nyquist plots of electrical impedance spectra of the PSCs based on pristine and 2FEABr treated $MAPbI_3$

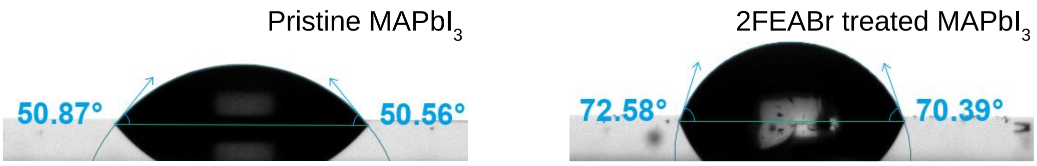

Fig. S16 Water contact angles of pristine and 2FEABr treated $MAPbI_3$ film

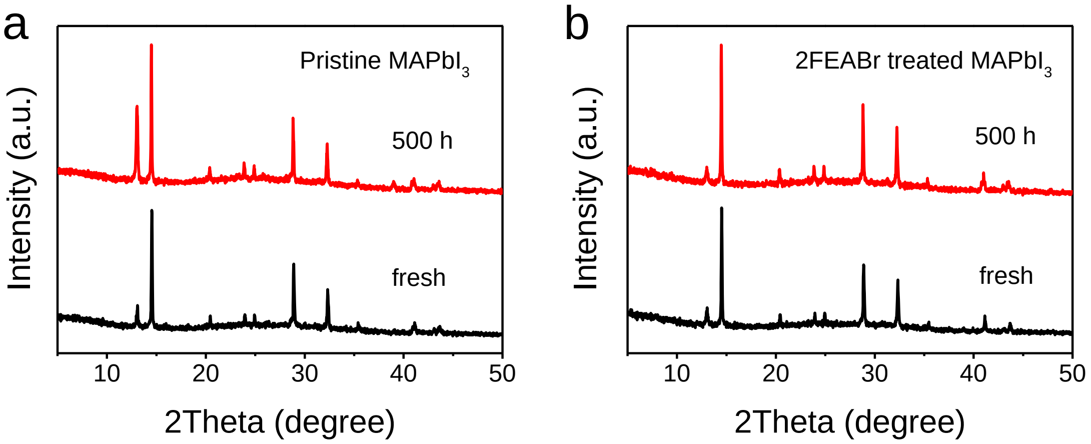

Fig. S17 The XRD patterns of pristine and 2FEABr treated $MAPbI_3$ films before and after 500 h calcination at 340 K

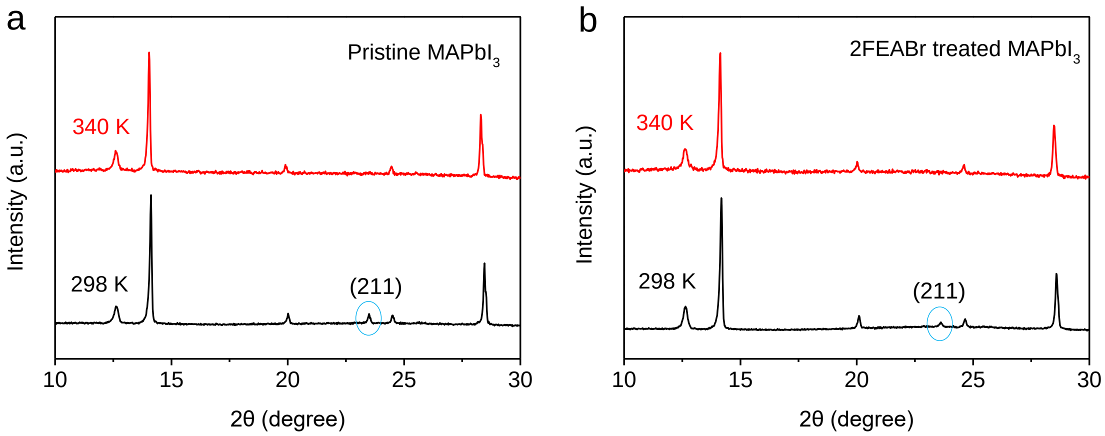

Fig. S18 XRD patterns of the pristine and 2FEABr treated $MAPbI_3$ films measured at 298 K and 340 K, respectively. The characterized peak at 23.5° is attributed to the (211) plane of tetragonal phase of $MAPbI_3$

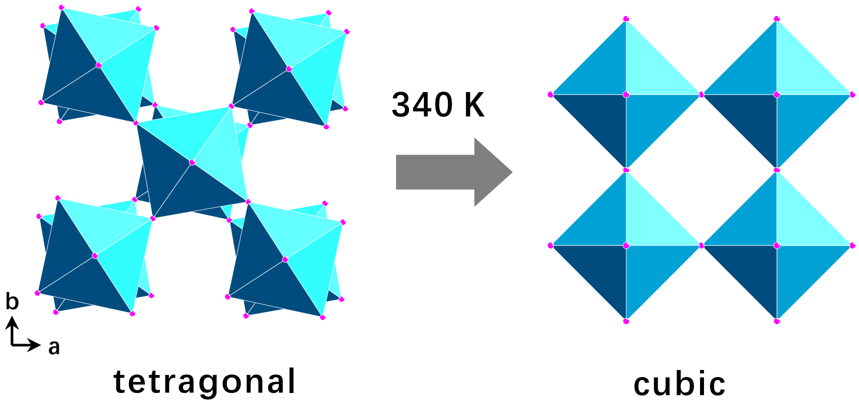

Fig. S19 Schematic illustration of phase transition for $MAPbI_3$ perovskite from tetragonal phase to cubic phase

Table S2 The fitted parameters of the TRPL spectra

	τ1 (ns)	A1 (%)	τ2 (ns)	A2 (%)
**Pristine**	20.5	72.9	101.8	27.1
**2FEABr treated**	32.3	68.4	145.6	31.6

Table S3 Photovoltaic parameters of the devices with different 2FEABr concentrations

2FEABr concentration	Voc (V)	Jsc (mA/cm2)	FF	PCE (%)
Pristine	1.090	22.38	0.797	19.44
1 mg/mL	1.148	22.49	0.802	20.69
2 mg/mL	1.166	22.39	0.807	21.06
3 mg/mL	1.165	21.79	0.799	20.28
5 mg/mL	1.175	20.38	0.771	18.47

Table S4 The fitting results of the equivalent circuit of Nyquist plots

PSCs	R_s (Ω)	R_{rec} (kΩ)	C_{rec} (nF)
**Pristine**	22	2.13	4.83
**2FEABr treated**	14	6.15	5.51
